# Supplementary material for: Sensory input drives rapid homeostatic scaling of the axon initial segment in mouse barrel cortex
Source: Nat Commun. 2021 Jan 4;12:23. doi: 10.1038/s41467-020-20232-x (PMC7782484; doi:10.1038/s41467-020-20232-x)
Supplement: Supplementary file 1 — Supplementary Information [file 41467_2020_20232_MOESM1_ESM.pdf]

Supplementary Information

Sensory input drives rapid homeostatic scaling of the axon initial segment in mouse barrel cortex

Nora Jamann, Dominik Dannehl, Nadja Lehmann, Robin Wagener, Corinna Thielemann, Christian Schultz, Jochen Staiger, Maarten H.P. Kole, Maren Engelhardt

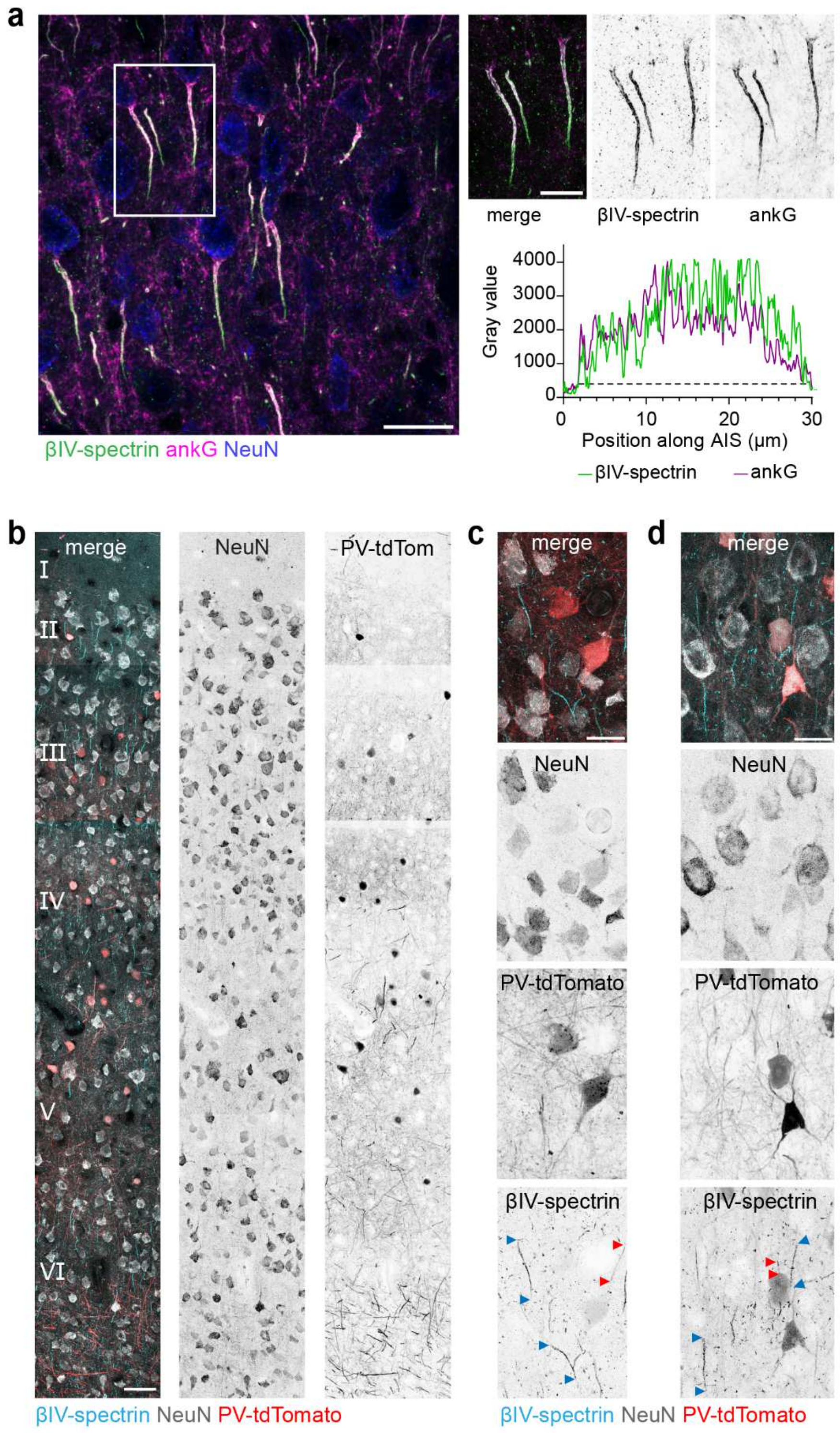

Fig. S1 Related to methods: Confirmation of antibody and cell population strategies

a *Left*: Double immunofluorescent staining for ankG (magenta) and ßIV-spectrin (green) was performed in mouse S1BF. Scale bar 20 µm. *Right*: Higher magnification of selected AIS (box in a) show the overlap between the two proteins, as expressed by the histogram plot of the immunofluorescent signal along the AIS. Dotted line indicates the 10% threshold of maximum fluorescence, which determines AIS beginning and end. The threshold is reached at the same location in both channels. Scale bar 10 µm.

b Panel of the entire cortex in S1BF of a parvalbumin - tdTomato (PV-tdtom) reporter mouse line, stained for NeuN (grey) and ßIV-spectrin (blue). Note the sparse occurance of interneurons (PV-positive). Cortical layers are indicated by Roman numerals. Scale bar 80 µm.

c, d Representative confocal images of AIS morphology of inhibitory and excitatory neurons stained for PV-tdTom (red), NeuN (grey) and ßIV-spectrin (cyan). Scale bar 20 µm. Note the typical pyramidal AIS running perpendicular to the cortical surface with a gradual decrease of AIS thickness from proximal to distal (blue arrowheads). Interneuron AIS are often oriented horizontally or towards the cortical surface and appear thin and faint (red arrowheads).

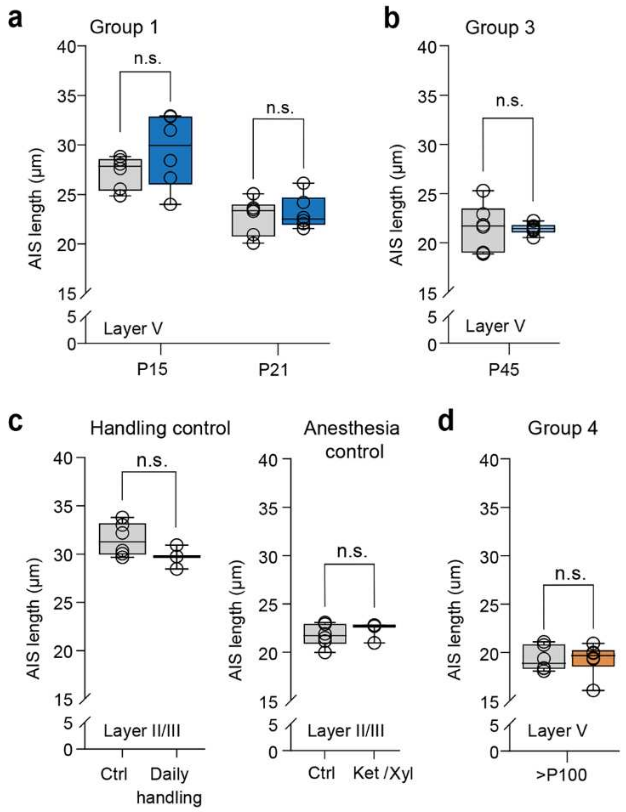

Figure S2. Related to Figure 2: Layer V AIS do not show activity-dependent plasticity

a Analysis of AIS length changes in layer V after long-term deprivation in group 1. At P15 and P21 no significant length changes were observed (Two-way ANOVA $P = 0.197$ for deprivation, $P < 0.0001$ for age, $P = 0.380$ for the interaction, Sidak's multiple comparisons $P > 0.05$, $n = 6$ biologically independent experiments).

b Analysis of AIS length changes in layer V after long-term deprivation in group 3. At P45 no significant length changes were observed (unpaired two-sided *t*-test $P = 0.879$, $n = 6$ biologically independent experiments)

c Control experiments for whisker trimming. *Left*: Equivalent to group 1, pups were handled daily from P0 to P15 but instead of trimming, whiskers were only slightly ruffled. No significant length changes were observed in this group (unpaired two-sided *t*-test $P = 0.1558$, $n = 3$ biologically independent experiments). *Right*: As an anesthesia control for group 4, adult mice were given a daily dose of ketamine/xylazine, however whiskers were not trimmed. In the anesthetized animals, no AIS length changes were observed as compared to adult controls (unpaired two-sided *t*-test $P = 0.625$, $n = 3$ biologically independent experiments).

d Analysis of AIS length changes in layer V after long-term deprivation in group 4. In adult mice, no significant length changes were observed (unpaired two-sided *t*-test $P = 0.95$, $n = 6$ biologically independent experiments)

a - d Boxplots indicate median with 25 to 75% interval and error bars show min. to max. values.

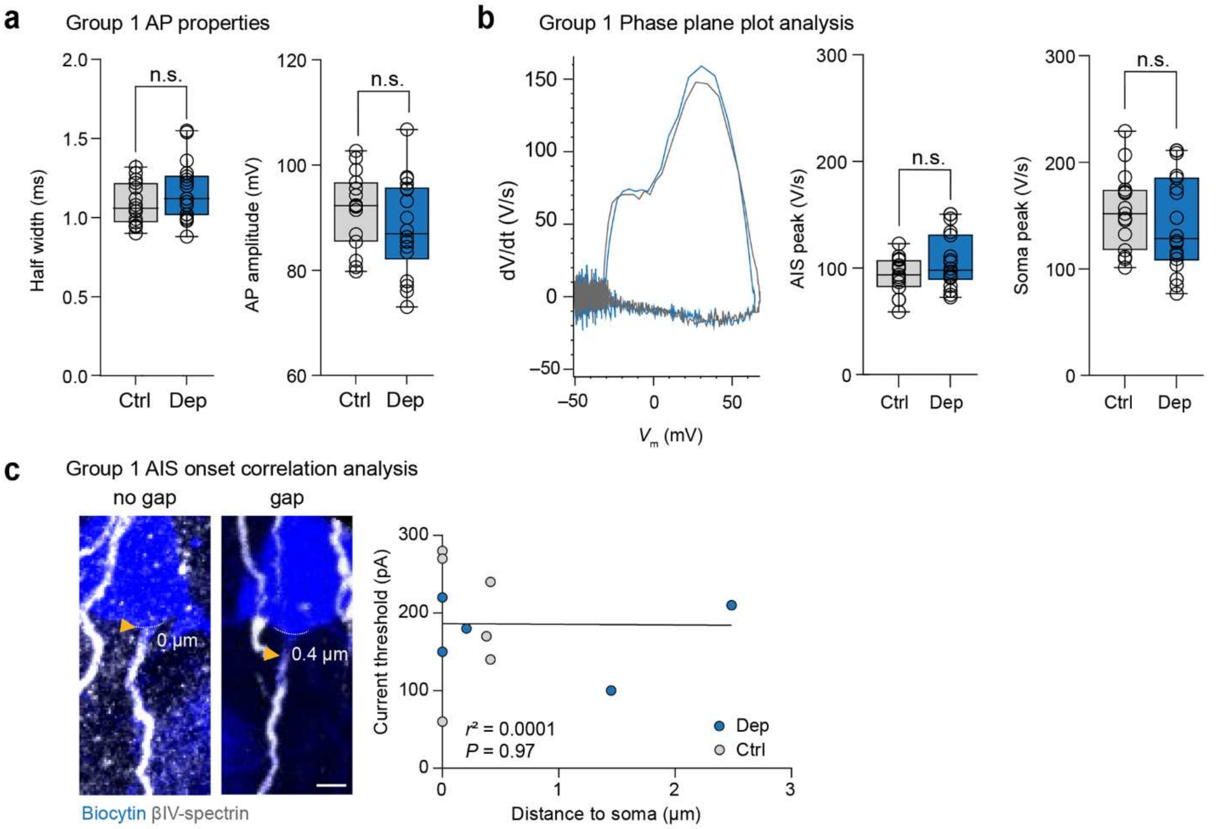

Figure S3. Related to Figure 3: AP waveform is conserved after sensory deprivation

a AP half width and AP Amplitude were not changed in group 1 after deprivation from P0 – P15 (unpaired two-sided *t*-test half width $P = 0.201$, AP amplitude P = 0.276, $n = 15$ cells for Ctrl, 20 cells for Dep from 7 biologically independent experiments).

b Phase plane plot analysis of Dep vs Ctrl APs. *Left*: Representative phase plane plots of a Ctrl and Dep neuron demonstrate the similarity in AP shape. *Right*: Analysis of the first and second peak (AIS and soma peak respectively) of the phase plane plot reveals no significant difference between deprivation and control neurons (unpaired two-sided *t*-test AIS peak $P = 0.106$, soma peak $P = 0.443$, $n = 18$ cells for Dep, $n = 15$ cells for Ctrl from 7 biologically independent experiments).

c Correlation analysis of AIS onset position (relative to the soma) and current threshold for Dep and Ctrl neurons. *Left*: Example of a neuron with no gap between soma and AIS vs. a neuron with a small gap. *Right*: There was no significant correlation of AIS onset position and current threshold. Results of linear regression analysis indicated in panel.

a, b Boxplots indicate median with 25 to 75% interval and error bars show min. to max. values.

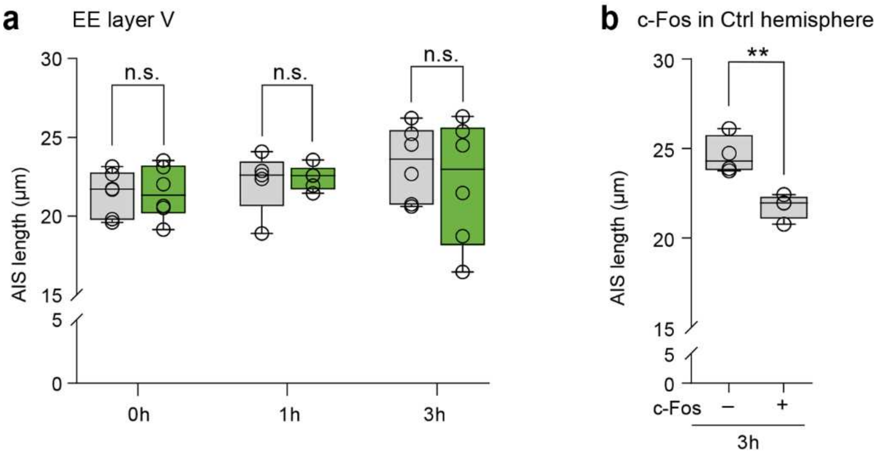

Figure S4. Related to Figure 4: Layer V AIS length remains unaltered after exposure to an enriched environment

a Analysis of AIS length changes after EE exposure in layer V. No significant length changes were observed after 1h and 3h of EE (Two-way ANOVA $P = 0.713$ for EE, $P = 0.40$ for time, $P = 0.715$ for the interaction, Sidak's multiple comparisons $P > 0.05$ for all comparisons, $n = 5$ (for 1 h) – 6 (for 0 h, 3 h) biologically independent experiments).

b C-Fos analysis of Ctrl hemisphere. After 3h of EE, analysis revealed a significantly decreased AIS length in c-Fos^{+} neurons (unpaired two-sided *t*-test, $^{**}P = 0.0046$, $n = 4$ biologically independent experiments, 6-14 c-Fos^{+} neurons/ animal, 20-50 c-Fos^{-} neurons/ animal).

a, b Boxplots indicate median with 25 to 75% interval and error bars show min. to max. values.

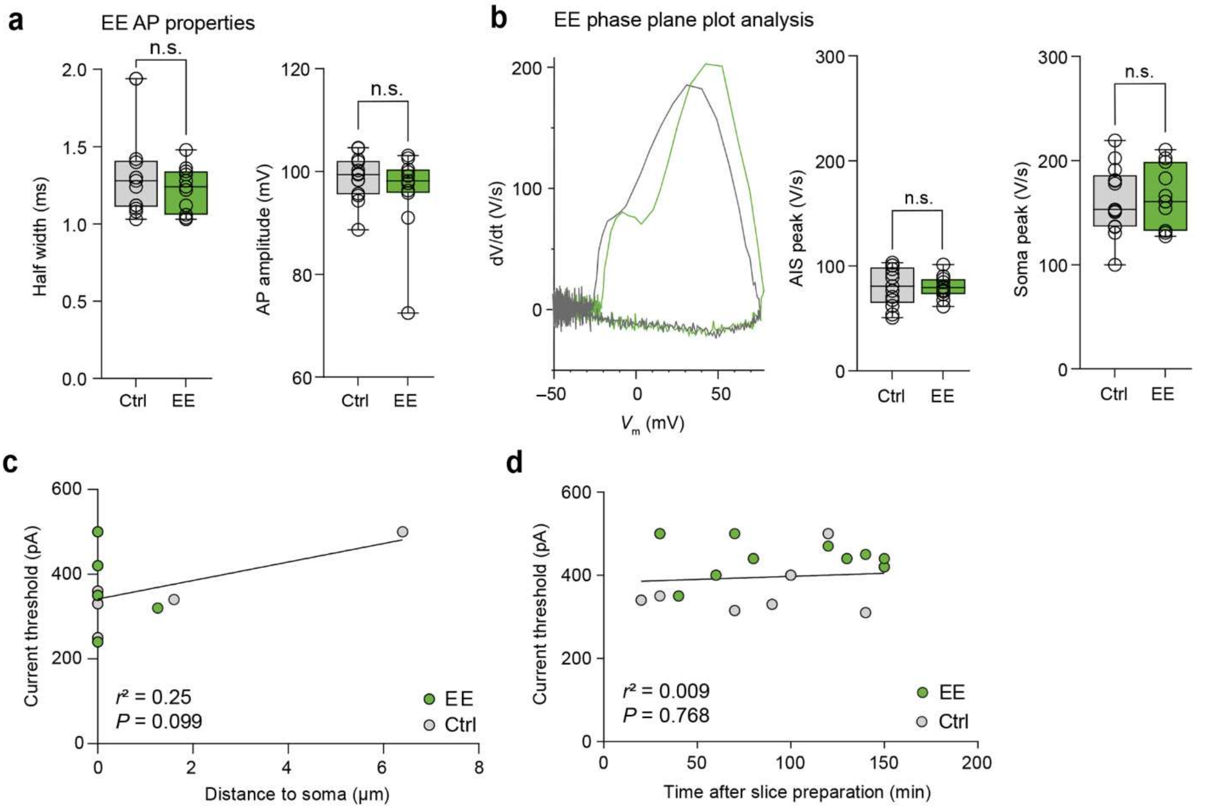

Figure S5. Related to Figure 5: AP waveform is conserved after exposure to an enriched environment

a AP half width and AP Amplitude were not changed after 3 h of EE (half width: Mann-Whitney test P = 0.578, AP amplitude: unpaired two-sided t-test P = 0.34, n = 13 cells for Ctrl, n = 11 cells for EE from 10 biologically independent experiments)

b Phase plane plot analysis of EE vs Ctrl APs. *Left*: Representative phase plane plots of a Ctrl and EE neuron demonstrate the similarity in AP shape. *Right*: Analysis of the first and second peak (AIS and soma peak respectively) of the phase plane plot reveals no significant difference between EE and control neurons (unpaired two-sided t-test AIS peak P = 0.783, soma peak P = 0.875, n = 10 cells EE, n = 13 cells Ctrl from 10 biologically independent experiments).

c Correlation analysis of AIS onset position (relative to the soma) and current threshold for EE and Ctrl neurons. There was no significant correlation of AIS onset position and current threshold. Results of linear regression analysis indicated in panel.

d Correlation analysis of the relationship between the time after slicing and the current threshold to control for reversibility of EE effect over time during slice incubation. The results of the linear regression analysis, as indicated in the panel, reveal no correlation between time after slice preparation and current threshold.

a, b Boxplots indicate median with 25 to 75% interval and error bars show min. to max. values.

Supplemental Tables

Table S1 *P*-values of multiple comparisons for AIS length during layer II/III development (Fig. 1b). **** indicates $P < 0.0001$. Significant results in red ($P < 0.05$).

vs.	E20	P1	P3	P7	P10	P13	P15	P21	P28	P35	P45	P180
**E20**												
**P1**	0.836											
**P3**	0.826	0.985										
**P7**	0.003	****	****									
**P10**	****	****	****	****								
**P13**	****	****	****	****	0.022							
**P15**	****	****	****	****	0.003	0.966						
**P21**	****	****	****	****	0.566	****	****					
**P28**	****	****	****	****	0.966	0.005	0.0006	0.823				
**P35**	****	****	****	****	0.966	0.003	0.0004	0.836	0.985			
**P45**	****	****	****	0.029	0.007	****	****	0.554	0.027	0.037		
**P180**	****	****	****	0.566	****	****	****	0.022	0.0004	0.0006	0.8005	

Table S2 *P*-values of multiple comparisons for AIS length during layer V development (Fig. 1b). **** indicates $P < 0.0001$. Significant results in red ($P < 0.05$).

vs.	E20	P1	P3	P7	P10	P13	P15	P21	P28	P35	P45	P180
**E20**												
**P1**	0.0004											
**P3**	****	0.918										
**P7**	****	****	0.01									
**P10**	****	****	****	0.003								
**P13**	****	****	0.0002	0.984	0.029							
**P15**	****	****	0.0005	0.984	0.014	0.984						
**P21**	****	0.528	0.984	0.056	****	0.002	0.004					
**P28**	****	0.373	0.984	0.096	****	0.004	0.0089	0.984				
**P35**	****	0.088	0.849	0.373	****	0.029	0.054	0.984	0.984			
**P45**	****	0.984	0.984	0.003	****	****	****	0.984	0.983	0.576		
**P180**	0.01	0.984	0.306	****	****	****	****	0.065	0.035	0.005	0.538	

Table S3 *P*-values of multiple comparisons for Western blot data (Fig. 1d). **** indicates $P < 0.0001$. Significant results in red ($P < 0.05$).

Comparison	AnkG 190 kDa	AnkG 270 kDa	AnkG 480 kDa
E20 vs. P1	0.9890	0.9930	0.8733
E20 vs. P3	0.9682	0.7633	0.6510
E20 vs. P7	>0.9999	0.9809	0.9155
E20 vs. P10	>0.9999	>0.9999	0.6225
E20 vs. P13	0.7608	0.3319	0.0486
E20 vs. P15	0.1882	0.0805	0.0013
E20 vs. P21	0.0650	0.0218	0.0302
E20 vs. P45	****	0.0002	0.0823
P1 vs. P3	>0.9999	0.9952	>0.9999
P1 vs. P7	0.9991	>0.9999	>0.9999
P1 vs. P10	0.9709	0.9791	0.9999
P1 vs. P13	0.2618	0.0785	0.5068
P1 vs. P15	0.0343	0.0148	0.0238
P1 vs. P21	0.0104	0.0038	0.3745
P1 vs. P45	****	****	0.6715
P3 vs. P7	0.9952	0.9988	0.9997
P3 vs. P10	0.9330	0.6698	>0.9999
P3 vs. P13	0.1970	0.0159	0.7577
P3 vs. P15	0.0240	0.0028	0.0553
P3 vs. P21	0.0072	0.0007	0.6186
P3 vs. P45	****	****	0.8868
P7 vs. P10	>0.9999	0.9544	0.9995
P7 vs. P13	0.5937	0.0591	0.4401
P7 vs. P15	0.1143	0.0109	0.0187
P7 vs. P21	0.0372	0.0028	0.3176
P7 vs. P45	****	****	0.6016
P10 vs. P13	0.8411	0.4155	0.7830
P10 vs. P15	0.2442	0.1091	0.0606
P10 vs. P21	0.0880	0.0303	0.6471
P10 vs. P45	****	0.0002	0.9040
P13 vs. P15	0.9634	0.9937	0.6905
P13 vs. P21	0.7354	0.8401	>0.9999
P13 vs. P45	0.0002	0.0242	>0.9999
P15 vs. P21	0.9995	0.9987	0.8199
P15 vs. P45	0.0020	0.1219	0.5259
P21 vs. P45	0.0066	0.3571	0.9998

Table S4 Summary of experimental groups in deprivation and enriched environment conditions.

Group	Deprivation period	End point	Control	Figure
1	P0-P15, *n* = 6 animals (IF)<br>P0-P15 Dep *n* = 20 cells from 7 animals; P15 control: 15 cells, from 7 animals (Ephys)	P15	P15	Fig. 2<br>Fig. 3
1	P0-P21, *n* = 6 animals (IF)	P21	P21	Fig. 2, S2
1	P0-P45, *n* = 6 animals (IF)	P45	P45	Fig. 2, S2
2	P0-P21, *n* = 6 animals (IF)	P45	P45	Fig. 2, S2
3	P10-P15, *n* = 6 animals (IF)	P15	P15	Fig. 2,
3	P10-P15, *n* = 6 animals (IF)	P21	P21	Fig. 2,
3	P10-P15, *n* = 6 animals (IF)	P45	P45	Fig. 2
4	16 days, *n* = 6 animals (IF)	> P100	> P100	Fig. 2, S2

Group	Enriched environment	End point	Control	Figure
5	0h, *n* = 6 animals (IF)	0h in EE<br>(12h after unilateral trimming)	0h cl hemisphere	Fig. 4
6	1 h, *n* = 5 animals (IF)	1 h in EE	1 h cl hemisphere	Fig. 4
7	3 h, *n* = 6 animals (IF)<br>3 h, *n* = 12 cells from 10 animals; control *n* = 13 cells from 10 animals (Ephys)	3 h in EE	3 h cl hemisphere	Fig. 4, S4<br>Fig. 5
8	6 h, *n* = 6 animals (IF)	6 h in EE	6 h cl hemisphere	Fig. 4
9	3 h EE, 3 h HC<br>*n* = 5 animals (IF)	6 h (3 h EE + 3h HC)	3 h EE + 3 h HC cl hemisphere	Fig. 4
10	6 h EE, 1 h new EE<br>*n* = 5 animals (IF)	7h (6 h EE + 1h EE)	6 h EE + 1h EE cl hemisphere	Fig. 4

IF immunofluorescence, *Ephys* electrophysiological recording, *EE* enriched environment, *cl* contralateral, *HC* home-cage

Table S5 Specification of antibodies (catalog number, working dilution, fixation of tissue, previously conducted controls, sources and references where available).

**Antibody**<br>**Clone/type**<br>**Catalog Number**	**Dilution**<br>**Applic.**	**Reported specificity**				**Source**<br>**Reference**<br>**RRID**
		KO	IF	IP	WB	
Ankyrin-G (rb)<br>H-215<br>sc-28561	1:500<br>4% PFA<br>1:1000<br>(WB)	X	X	X	X	Santa Cruz, Heidelberg, Germany<br>[1]<br>AB_633909
Ankyrin-G (ms)<br>N106/36<br>73-146	1:500<br>4% PFA	X	X		X	UC Davis/NIH NeuroMab Facility, CA, USA<br>[1]<br>AB_2315803
ßIV-spectrin (rb)<br>amino acids 2237-2256 of human ßIV-spectrin	1:500<br>4% PFA<br>1:2000<br>(WB)	X	X		X	Selfmade<br>[2, 3, 4]
NeuN (ms)<br>A60<br>MAB377	1:500<br>4% PFA		X		X	Millipore, Temecula, USA<br>[1, 2]
NeuN (gp)<br>*ABN90*	1:2000<br>4% PFA		X			Merck Millipore, Darmstadt, Germany<br>[5]
Actin (rb)<br>*I-19*<br>*sc-1616-R*	1:5000<br>WB					Santa Cruz, Heidelberg, Germany
c-Fos (rb)<br>*9F6*<br>*#2250*	1:400<br>4% PFA		X		X	Cell Signaling, Frankfurt am Main, Germany
gt anti ms Alexa Fluor 488; A28175	1:1000					Molecular Probes, Thermo Fisher, Karlsruhe, Germany<br>AB_2535764
gt anti rb Alexa Fluor 488; A32731	1:1000					Molecular Probes, Thermo Fisher, Karlsruhe, Germany<br>AB_143165
gt anti gp Alexa Fluor 568; A32723	1:1000					Molecular Probes, Thermo Fisher, Karlsruhe, Germany<br>AB_2534119
gt anti ms Alexa Fluor 568; A11004	1:1000					Molecular Probes, Thermo Fisher, Karlsruhe, Germany<br>AB_143162
gt anti rb Alexa Fluor 514; A31558	1:1000					Molecular Probes, Thermo Fisher, Karlsruhe, Germany<br>AB_2536173
Alexa Streptavidin 568; S11226	1:1000					Molecular Probes, Thermo Fisher, Karlsruhe, Germany<br>AB_2315774

KO absence of immunostainings in knock out animals, *IF* immunofluorescence, *IP* immuno-precipitation, *WB* western blot, *rb* rabbit, *ms* mouse, *gp* guinea pig

Table S6 Summary of additional control groups for deprivation experiments

Group	Treatment	End Point	IF	Figure
1	P0-P15 only handling, no trimming	P15	*n* = 3	S2D
2	16 days, only anesthesia, no trimming	>P100	*n* = 3	S2D

IF = immunofluorescence

References for supplements

1. Schlüter A, Del Turco D, Deller T, Gutzmann A, Schultz C, Engelhardt M. Structural Plasticity of Synaptopodin in the Axon Initial Segment during Visual Cortex Development. *Cereb Cortex* **27**, 4662-4675 (2017).

2. Gutzmann A, Ergul N, Grossmann R, Schultz C, Wahle P, Engelhardt M. A period of structural plasticity at the axon initial segment in developing visual cortex. *Front Neuroanat* **8**, 11 (2014).

3. Freal A*, et al.* Cooperative Interactions between 480 kDa Ankyrin-G and EB Proteins Assemble the Axon Initial Segment. *J Neurosci* **36**, 4421-4433 (2016).

4. Höfflin F*, et al.* Heterogeneity of the Axon Initial Segment in Interneurons and Pyramidal Cells of Rodent Visual Cortex. *Front Cell Neurosci* **11**, 332 (2017).

5. Benedetti B, Dannehl D, Janssen JM, Corcelli C, Couillard-Despres S, Engelhardt M. Structural and Functional Maturation of Rat Primary Motor Cortex Layer V Neurons. *Int J Mol Sci* **21**, (2020).
