# Supplementary material for: Sensory input drives rapid homeostatic scaling of the axon initial segment in mouse barrel cortex
Source: Nat Commun. 2021 Jan 4;12:23. doi: 10.1038/s41467-020-20232-x (PMC7782484; doi:10.1038/s41467-020-20232-x)
Supplement: Supplementary file 3 — Reporting Summary [file 41467_2020_20232_MOESM3_ESM.pdf]

nature research

Corresponding author(s): Maren Engelhardt

Last updated by author(s): Nov 11, 2020

Reporting Summary

Nature Research wishes to improve the reproducibility of the work that we publish. This form provides structure for consistency and transparency in reporting. For further information on Nature Research policies, see our Editorial Policies and the Editorial Policy Checklist.

Statistics

For all statistical analyses, confirm that the following items are present in the figure legend, table legend, main text, or Methods section.

n/a	Confirmed	
☐	☒	The exact sample size (n) for each experimental group/condition, given as a discrete number and unit of measurement
☐	☒	A statement on whether measurements were taken from distinct samples or whether the same sample was measured repeatedly
☐	☒	The statistical test(s) used AND whether they are one- or two-sided<br>*Only common tests should be described solely by name; describe more complex techniques in the Methods section.*
☐	☒	A description of all covariates tested
☐	☒	A description of any assumptions or corrections, such as tests of normality and adjustment for multiple comparisons
☐	☒	A full description of the statistical parameters including central tendency (e.g. means) or other basic estimates (e.g. regression coefficient) AND variation (e.g. standard deviation) or associated estimates of uncertainty (e.g. confidence intervals)
☐	☒	For null hypothesis testing, the test statistic (e.g. F, t, r) with confidence intervals, effect sizes, degrees of freedom and P value noted<br>*Give P values as exact values whenever suitable.*
☒	☐	For Bayesian analysis, information on the choice of priors and Markov chain Monte Carlo settings
☒	☐	For hierarchical and complex designs, identification of the appropriate level for tests and full reporting of outcomes
☐	☒	Estimates of effect sizes (e.g. Cohen's d, Pearson's r), indicating how they were calculated

Our web collection on statistics for biologists contains articles on many of the points above.

Software and code

Policy information about availability of computer code

Data collection	The following software were used to aquire and collect data (latest version if not specified otherwise):<br>Confocal images: NIS Elements (Nikon Instruments); LAS X Life Science (Leica Microsystems)<br>Electrophysiological data: PatchMaster 2x90 (HEKA Electronics)<br>Western blot: Fusion solo s (Vilber Lourmat)
Data analysis	The following software was used to analyse data (latest version if not specified otherwise):<br>Image processing: FIJI (ImageJ, no version number available), Adobe Photoshop CC 2018 (Adobe Systems)<br>Morphometrical analyses: FIJI (ImageJ, no version number available), AISuite (https://github.com/jhnnsrs/aisuite2, version 2)<br>Western Blot: FIJI (ImageJ, no version number available)<br>Electrophysiological data: FitMaster 2x90 (HEKA Electronics), OriginPro 8 (Origin lab Corporation), AxoGraph X (AxoGraph Scientific)<br>Statistical analysis: GraphPad Prism 8 (GraphPad Software, Inc.)

For manuscripts utilizing custom algorithms or software that are central to the research but not yet described in published literature, software must be made available to editors and reviewers. We strongly encourage code deposition in a community repository (e.g. GitHub). See the Nature Research guidelines for submitting code & software for further information.

Data

Policy information about availability of data

All manuscripts must include a data availability statement. This statement should provide the following information, where applicable:

- Accession codes, unique identifiers, or web links for publicly available datasets
- A list of figures that have associated raw data
- A description of any restrictions on data availability

The data that support the findings of this study are available from the corresponding author upon reasonable request.

Field-specific reporting

Please select the one below that is the best fit for your research. If you are not sure, read the appropriate sections before making your selection.

☒ Life sciences ☐ Behavioural & social sciences ☐ Ecological, evolutionary & environmental sciences

For a reference copy of the document with all sections, see nature.com/documents/nr-reporting-summary-flat.pdf

Life sciences study design

All studies must disclose on these points even when the disclosure is negative.

Sample size	Sample sizes were chosen based on previous studies performed in our lab (Gutzmann et al., 2014, Schlüter et al., 2017, Schlüter et al., 2019), where a certain sample size generated a dataset for analysis with sufficent power (P > 0.8). This sample size was usually a minimum n = 5 mice (in most cases 6) for immunohistochemistry in all experimental and control groups; in each animal, a set of at least 100 individual datapoints (for each AIS) were analyzed. For electrophysiology, a minimum n = 10 cells were included in each experimental and control group (Benedetti et al., 2020).
Data exclusions	No data was excluded.
Replication	Replication was achieved when considering individual mice (with at least 100 data points analyzed per mouse) as individual samples, with 5-6 mice per experimental and control group (each mouse would be a replication within one experimental group). In the light of the 3R, we did not replicate entire experimental groups (6 mice per group; replication would have resulted in at least double, if not triple the amount of animals used). In the light of our careful statistical analysis, we see no justification for such excessive testing.
Randomization	Mice from individual litters (both genders) were randomly assigned to experimental or control groups.
Blinding	Both the morphometrical as well as the electrophysiological data were analyzed in a standardized, bias-free fashion (e.g. determining start and end of the axon initial segment based on a software-determined threshold, not the experimentor's subjective visual impression). Therefore, data was not blinded. For in vitro experiments, investigators were blinded to conditions.

Reporting for specific materials, systems and methods

We require information from authors about some types of materials, experimental systems and methods used in many studies. Here, indicate whether each material, system or method listed is relevant to your study. If you are not sure if a list item applies to your research, read the appropriate section before selecting a response.

Materials & experimental systems

n/a	Involved in the study	
☐	☒	Antibodies
☒	☐	Eukaryotic cell lines
☒	☐	Palaeontology and archaeology
☐	☒	Animals and other organisms
☒	☐	Human research participants
☒	☐	Clinical data
☒	☐	Dual use research of concern

Methods

n/a	Involved in the study	
☒	☐	ChIP-seq
☒	☐	Flow cytometry
☒	☐	MRI-based neuroimaging

Antibodies

Antibodies used	Name (species), Clone/type, Catalog Number, Source, Research Resource Identifier (RRID)where available:<br>Ankyrin-G (rb), H-215, sc-28561, Santa Cruz, Heidelberg, Germany, AB_633909<br>Ankyrin-G (ms), N106/36, 73-146, UC Davis/NIH NeuroMab Facility, CA, USA, AB_2315803<br>ßIV-spectrin (rb), amino acids 2237-2256 of human ßIV-spectrin, Selfmade (published previously)<br>NeuN (ms), A60, MAB377, Millipore, Temecula, CA, USA

NeuN (gp), 266 004, Synaptic Systems GmbH, Göttingen, Germany
Actin (rb), I-19, sc-1616-R, Santa Cruz, Heidelberg, Germany
c-Fos (rb), 9F6, #2250, Cell Signaling, Frankfurt am Main, Germany

Secondary antibodies all from Molecular Probes, Thermo Fisher, Karlsruhe, Germany:
gt anti ms Alexa Fluor 488; A28175, AB_2535764
gt anti rb Alexa Fluor 488; A32731, AB_143165
gt anti gp Alexa Fluor 568; A32723, AB_2534119
gt anti ms Alexa Fluor 568; A11004, AB_143162
gt anti rb Alexa Fluor 514; A31558, AB_2536173
Alexa Streptavidin 568; S11226, AB_2315774

The paper further lists the nuclear stain TO-PRO-3 iodide in the context of antibodies, but this reagent is not an antibody, hence we have removed it from the antibody table, and included the information in the Methods section on immunostaining.

Validation

Specificity tested via knockout (KO), immunofluorescence (IF), immunoprecipitation (IP), or Western Blot (WB) as outlined in the antibody table in the Supplementary material. Species included below.

Ankyrin-G (rb): KO, IF, IP, WB
Ankyrin-G (ms): KO, IF, WB
ßIV-spectrin (rb): KO, IF, WB
NeuN (ms): IF, WB
NeuN (gp): IF
Actin (rb): -
c-Fos (rb): IF, WB

Animals and other organisms

Policy information about studies involving animals; ARRIVE guidelines recommended for reporting animal research

Laboratory animals

All experiments were conducted using wildtype mice (C57BL/6JRj obtained from Janvier Labs, France). Animals were randomly picked for the different experimental and control groups, and all groups consisted of animals from both genders. All animals were maintained with food and water ad libitum on a regular 12 h light/dark cycle in ambient light conditions (300 lux during light, 25 lux in the resting section of all cages). Humidity was kept at 50% (+/- 10 %) and room temperature was 22° C (+/- 2° C). A comprehensive list of all experimental and control age groups, as well as deprivation details, is provided in Table 1 of the main manuscript.

Wild animals

This study did not involve wild animals.

Field-collected samples

This study did not involve field-collected samples.

Ethics oversight

All animal procedures were carried out in accordance with the recommendations of the Animal Research Council of the Medical Faculty Mannheim, Heidelberg University and were approved by the State of Baden-Württemberg and compliant with EU guidelines (protocol numbers 35-9185.81/G-242/12 and 35-9185.81/G-290/16). The revision of the manuscript required a new animal protocol permit, which we acquired from the appropriate State institution (35-9185.81/G-119/20).

Note that full information on the approval of the study protocol must also be provided in the manuscript.
